# Safety and Efficacy of Ivermectin for the Prevention and Treatment of COVID-19: A Double-Blinded Randomized Placebo-Controlled Study

**DOI:** 10.3390/antibiotics11060796

**Published:** 2022-06-12

**Authors:** Nasikarn Angkasekwinai, Pinyo Rattanaumpawan, Methee Chayakulkeeree, Pakpoom Phoompoung, Pornpan Koomanachai, Sorawit Chantarasut, Walaiporn Wangchinda, Varalak Srinonprasert, Visanu Thamlikitkul

**Affiliations:** 1 Division of Infectious Diseases and Tropical Medicine, Department of Medicine, Faculty of Medicine Siriraj Hospital, Mahidol University, 2 Wanglang Road, Bangkoknoi, Bangkok 10700, Thailand; pinyo.rat@mahidol.ac.th (P.R.); methee.cha@mahidol.ac.th (M.C.); pakpoom.pho@mahidol.ac.th (P.P.); pornpan.koo@mahidol.ac.th (P.K.); walaiporn.wan@mahidol.ac.th (W.W.); visanu.tha@mahidol.ac.th (V.T.); 2 Department of Medicine, Faculty of Medicine Siriraj Hospital, Mahidol University, 2 Wanglang Road, Bangkoknoi, Bangkok 10700, Thailand; sorawit.cha@mahidol.ac.th; 3 Division of Geriatric Medicine, Department of Medicine, Faculty of Medicine Siriraj Hospital, Mahidol University, 2 Wanglang Road, Bangkoknoi, Bangkok 10700, Thailand; varalak.sri@mahidol.ac.th; 4 Siriraj Research Data Management Unit (Si-RDMU), Department of Research, Faculty of Medicine Siriraj Hospital, Mahidol University, 2 Wanglang Road, Bangkoknoi, Bangkok 10700, Thailand

**Keywords:** COVID-19, ivermectin, randomized-controlled trial, prevention, treatment

## Abstract

The safety and efficacy of ivermectin for the prevention and treatment of COVID-19 are still controversial topics. From August to November 2021, we conducted a double-blinded, randomized controlled trial at Siriraj Hospital, Thailand. Eligible participants were adults ≥ 18 years with suspected COVID-19 who underwent a SARS-CoV-2 RT-PCR test. After enrollment, the participants were randomized to receive either ivermectin (400–600 µg/kg/d) or placebo once daily for 3 days. Among 983 participants, 536 (54.5%) with a negative RT-PCR result were enrolled in the prevention study, and 447 (45.5%) with a positive RT-PCR result were enrolled in the treatment study. In the prevention study, the incidence of COVID-19 on Day 14 was similar between the ivermectin and the placebo group (4.7% vs. 5.2%; *p* = 0.844; Δ = −0.4%; 95% CI; −4.3–3.5%). In the treatment study, there was no significant difference between the ivermectin and placebo group for any Day 14 treatment outcome: proportion with oxygen desaturation (2.7% vs. 1.9%; *p* = 0.75), change in WHO score from baseline (1 [−5, 1] vs. 1 [−5, 1]; *p* = 0.50), and symptom resolution (76% vs. 82.2%; *p* = 0.13). The ivermectin group had a significantly higher proportion of transient blurred vision (5.6% vs. 0.6%; *p* < 0.001). Our study failed to demonstrate the efficacy of a 3-day once daily of ivermectin for the prevention and treatment of COVID-19. The given regimen of ivermectin should not be used for either prevention or treatment of COVID-19 in populations with a high rate of COVID-19 vaccination.

## 1. Background

The coronavirus disease 2019 (COVID-19) pandemic, caused by severe acute respiratory syndrome coronavirus 2 (SARS-CoV-2), remains a major health threat, with almost 440 million confirmed cases and six million deaths globally as of 1 March 2022 [1]. Effective vaccines are an essential measure to limit the COVID-19 pandemic; however, breakthrough infections and the continuation of the pandemic might occur owing to the emergence of new SARS-CoV-2 variants of concern [2]. The availability of effective antiviral treatments remains limited. Repurposing existing medicines that are readily available and inexpensive is therefore of great interest [3].

Ivermectin, an oral antiparasitic agent with broad-spectrum antiviral activity, has shown potent in vitro anti-SARS-CoV-2 activity; it induced a 5000-fold reduction in viral RNA after 48 h, with a half-maximal inhibitory concentration (IC_50_) of 2 µM [4]. Because of its several mechanisms for potential antiviral and anti-inflammatory activity [5], ivermectin has been evaluated in many studies for the treatment of SARS-CoV-2 infection, with high doses of up to 2 mg/kg and courses of up to 4 days [6,7]. However, most previous studies on the efficacy of ivermectin to treat COVID-19 were non-randomized and open-label, performed in settings with limited access to COVID-19 vaccines [8,9,10]. Furthermore, there were only few studies that focused on the efficacy of ivermectin for prevention of SARS-CoV-2 infection [11]. To determine the efficacy of ivermectin in preventing the acquisition of SARS-CoV-2 among a high-risk exposure population, and to evaluate the efficacy of ivermectin for treating laboratory-confirmed COVID-19, we performed a pragmatic randomized, placebo-controlled trial comparing a 3-day once daily dose of ivermectin with a placebo in an outpatient setting.

## 2. Methods

### 2.1. Study Design and Patients

This was a single-center, double-blinded, pragmatic randomized placebo-controlled trial conducted from August to November 2021 at Siriraj Hospital, a 2300-bed university hospital in Bangkok, Thailand. The study protocol was approved by the Siriraj Institutional Review Board (certificate of approval no. Si 607/2021) and was conducted in accordance with the Declaration of Helsinki. Written informed consent was obtained from all participants.

Participants were eligible for inclusion if they were ≥18 years and were suspected of having SARS-CoV-2 infection because of their respiratory tract symptoms or because they had a history of contact with a confirmed COVID-19 patient. Eligible participants also must have had a documented positive or negative test for SARS-CoV-2 (RT-PCR) from a nasopharyngeal (NP) swab sample taken on the enrollment day. Participants were excluded if they were pregnant or breastfeeding, had a history of ivermectin hypersensitivity, had a previous SARS-CoV-2 infection within 3 months, or had an inconclusive result on their RT-PCR SARS-CoV-2 test.

### 2.2. Randomization and Masking

Participants were randomized in a 1:1 ratio to receive standard of care plus ivermectin (Atlantic Laboratory Ltd., Bangkok, Thailand) or an identical placebo. Randomization was performed by using the computer-generated method with a varying block size of 2 to 8. Only the pharmacist knew the treatment assignment. The participants and investigators were blinded to the treatment assignment for the entire study period.

### 2.3. Interventions

Participants were given either placebo or ivermectin based on their body weight; the ivermectin dose ranged from 400–600 µg/kg/d. The dosage was calculated to the nearest 6 mg or 12 mg whole tablets (dosing table in the study protocol, Supplement File S1). The participants were advised to take the study medication before a meal on the enrollment day (Day 0) and once every 24 h for 2 consecutive days. After the RT-PCR result was available (within the same day), the participants with a negative result were included into the prevention study, while those with a positive result were included into the treatment study.

### 2.4. Procedures

Participants in the prevention study were instructed to collect an NP swab for the rapid detection of SARS-CoV-2 antigen using the Standard Q COVID-19 Ag test (SD Biosensor, Inc., Gyeonggi-do, Yongin-si, Korea) on Day 14 and whenever they developed new symptoms suggestive of COVID-19. If the rapid antigen test was positive, NP swab sampling for RT-PCR testing was performed at the hospital. Participants in the treatment study were instructed to measure their temperature and oxygen saturation on Day 3, Day 7, and Day 14. In accordance with the Thailand National Clinical Practice Guidelines for the Treatment of COVID-19, favipiravir was recommended for all symptomatic patients and all asymptomatic patients with risk factors for disease progression.

All participants were contacted by telephone on Day 3, Day 7, and Day 14 to collect data on temperature, oxygen saturation, symptoms, and safety of study medication.

### 2.5. Outcome Measurement

The primary outcomes of both prevention and treatment studies were analyzed using intention to treat (ITT) and modified intention to treat (mITT) populations. The ITT population comprised all eligible participants who were randomized and applied a worse-case scenario. All participant without evaluable outcomes and drop-out participant were considered as having a poor outcome. The mITT population included all randomized participants who received at least one dose of study drug. Participants in the prevention study who did not perform a second NP swab within 14 days were assumed to have a negative RT-PCR result in the mITT population if they were asymptomatic on Day 28 without proof of a RT-PCR test taken elsewhere.

The primary outcome of the prevention study was the proportion of participants with a positive RT-PCR within 14 days after enrollment among those with a negative RT-PCR result at enrollment in the mITT population. The primary outcomes of the treatment study were the proportion of participants with oxygen desaturation (oxygen saturation < 96% or decreased from baseline by ≥3% after exertion); changes in the WHO 10-point clinical progression score [12] on Day 3, Day 7, and Day 14 compared to baseline; the absence of all symptoms at Day 3, Day 7, and Day 14; hospitalization within 14 days; and 28-day mortality in the mITT population.

The secondary outcome of the study was the safety of the study medications, including the number and the percentage of participants with adverse effects (AEs) evaluated in the mITT population.

### 2.6. Sample Size

For the primary outcomes of the prevention study, we anticipated that a 3-day course of ivermectin would reduce the rate of SARS-CoV-2 infection from 20% to 10%. To achieve a power of 80% and a two-sided *p*-value of 0.05, 199 participants/group were required. Considering potential dropouts, a total of 478 participants with a negative RT-PCR at the enrollment were needed.

For the primary outcome of the treatment study, it was assumed that ivermectin would reduce the rate of oxygen desaturation of COVID-19 patients from 30% to 15%. To achieve 80% power and a two-sided *p*-value of 0.05, 121 participants/group were required. Considering potential dropouts, 290 participants with a positive RT-PCR at the enrollment were needed.

Given that the prevalence of SARS-CoV-2 infection among the patients who visited the acute respiratory tract infection (ARI) clinic was 50%, we needed to enroll at least 1000 patients who presented to the ARI clinic to achieve the target sample size for both studies.

### 2.7. Statistical Analysis

Demographic and baseline characteristics are presented as descriptive statistics. Continuous data are presented as the mean (standard deviation) or median (range), as appropriate. Categorical data are presented as number and percentage. The unpaired *t*-test and Mann–Whitney U test were used to compare continuous data, while the chi-square test or Fisher’s exact test was used to compare categorical data as appropriate. All statistical analyses were performed with PASW Statistics (SPSS) 18.0 (IBM Corp., Armonk, NY, USA). A *p*-value < 0.05 was considered statistically significant.

## 3. Results

**Figure 1:**
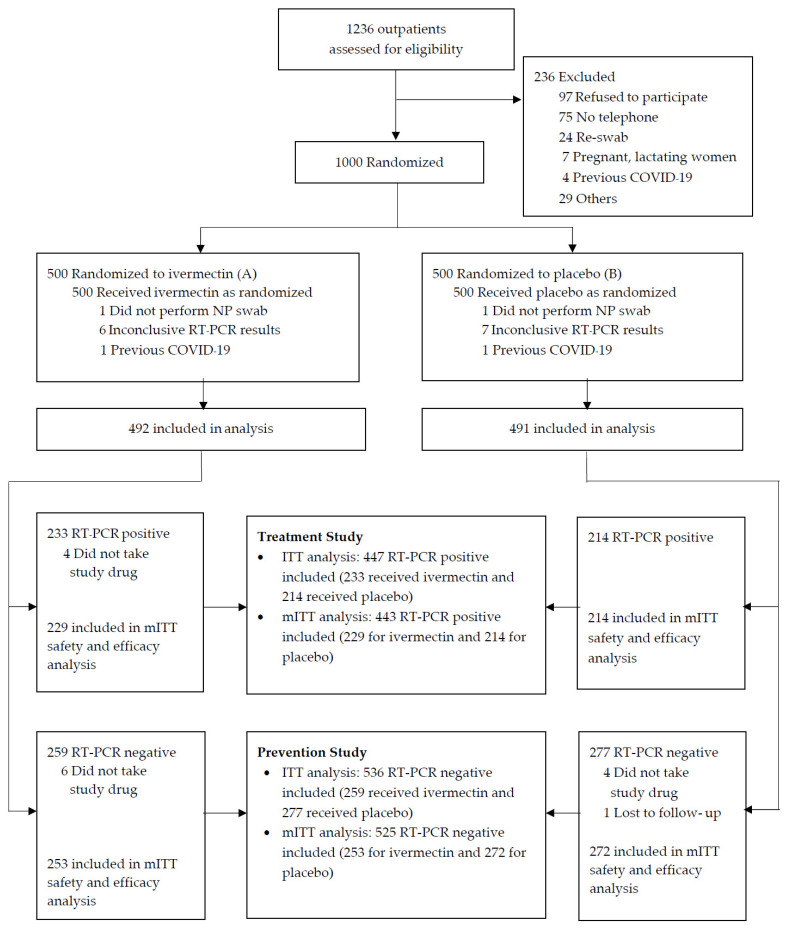
Enrollment, randomization, and treatment assignment.

Among 1236 patients who were screened from August 2021–October 2021, 1000 were recruited for the study; 500 were randomized to receive ivermectin and 500 to receive placebo. Seventeen participants (1.7%) were excluded owing to the pre-specified exclusion criteria. Among 983 participants, 968 (98.5%) completed the 28-day follow-up (Figure 1). The baseline information and clinical characteristics of the 983 participants were similar between two groups (Table S1). The mean age of all participants was 38.4 ± 12.1 years, 57.4% were female, and 30.6% had pre-existing diseases. Overall, 80% of the participants had previously received ≥1 dose of a COVID-19 vaccine. Of the 983 participants, 536 (54.5%) with a negative RT-PCR SARS-CoV-2 test were included in the prevention study, and 447 (45.5%) with a positive RT-PCR were included in the treatment study (Figure 1).

### 3.1. Primary Outcome of Ivermectin Prevention Study

Among the 536 participants with a negative RT-PCR result at enrollment, 259 were in the ivermectin group and 277 were in the placebo group. The baseline and clinical characteristics of the participants in both groups were similar (Table 1). The mean age was 37.6 ± 12.0 years, 57.8% were female, and 29.5% had pre-existing diseases. Approximately 90% of the participants had exposure risk, mainly a household contact with confirmed COVID-19, within 7 days before their RT-PCR test. Nearly 40% of participants were asymptomatic, and most (85%) had previously received ≥1 dose of a COVID-19 vaccine. Of the 536 participants, 11 participants were excluded from the mITT analysis because of various reasons. Therefore, 525 participants (253 in the ivermectin group and 272 in the placebo group) were included in the mITT analysis (Figure 1). Three participants in the ivermectin group and two participants in the placebo group did not perform follow-up NP swab testing for SARS-CoV-2 detection. The proportion of positive RT-PCR within 14 days was similar in the ivermectin and placebo groups for the ITT analysis (6.95% vs. 6.86%, *p* = 1.000), with a difference of −0.09% [95%CI, −4.3–4.6%]. The proportions were also similar in the mITT analysis (4.74% vs. 5.15%, *p* = 0.844), with a difference of −0.41% [95%CI, −4.3–3.5%] (Table 2). The median time to a positive RT-PCR test was 6 days, and there was no significant difference between the groups. In the mITT population subgroup analyses, there were no differences in the proportion of participants with a positive RT-PCR when analyzed by the contact duration, body weight, and vaccination status (Table S2).

### 3.2. Primary Outcomes of Ivermectin Treatment Study

Among the 447 participants with a positive RT-PCR at enrollment, 233 were in the ivermectin group and 214 were in the placebo group. The baseline and clinical characteristics were similar in the groups (Table 3). The mean age was 39.5 ± 12.1 years, 56.8% were female, and 32% had pre-existing diseases. Approximately 88% of participants had ≥1 symptom, of which cough (50.6%), sore throat (47%), and fever (38%) were the most frequent. Overall, 55.6% of participants had onset of symptoms ≤ 3 days before enrollment, and 60% of participants had a cycle threshold (Ct) value ≤ 20. Overall, 21.5% of the participants were COVID-19 vaccine-naive. Almost all (97.5%) received favipiravir concomitantly with the study medication. Four participants in the ivermectin group were excluded because they did not take the drug. Therefore, 443 participants (229 in the ivermectin group and 214 in the placebo group) were included in the mITT analysis (Figure 1).

For both the ITT and mITT analyses, there were no significant differences between ivermectin (plus favipiravir) and the placebo (plus favipiravir) for all outcomes, including the proportion of participants with oxygen desaturation; the change in WHO progression score from baseline; the absence of symptoms at Day 3, Day 7, and Day 14; 14-day hospitalization rate; and 28-day mortality (Table 4). Most symptoms gradually subsided over time except for loss of smell, which showed a peak frequency on Day 3 (Figure S1). In the mITT population, subgroup analysis did not reveal any differences in outcomes between the ivermectin group and the placebo group (Table S3). No participants died in this study. One participant in the ivermectin group and one participant in the placebo group reported COVID-19 infection on Day 23 and Day 17, respectively. In addition, no factors associated with favorable outcomes in participants who had an absence of all symptoms on Day 7 could be identified (Table S4).

### 3.3. Adverse Events

The incidences of AEs in participants in both groups are shown in Table 5. There was no significant difference in the proportion of participants reporting AEs between the ivermectin and placebo groups (21.6% vs. 18.9%, *p* = 0.337). However, there were more ocular AEs reported in the ivermectin group (5.6% vs. 0.6%, *p* < 0.001). These were mainly blurred vision while taking ivermectin, but this spontaneously resolved after completing the medication. An analysis of AEs by the study cohort found that ocular problems were more prevalent in the ivermectin group than the placebo group in the treatment cohort (8.7% vs. 0%, *p* < 0.001). Headache was reported more often in the placebo group (4.5% vs. 1.9%, *p* = 0.027) (Table S5). No serious AEs were reported in this study.

## 4. Discussion

To the best of our knowledge, this was the first large, double-blinded, randomized controlled trial to determine the safety and efficacy of ivermectin for both the treatment and prevention of COVID-19 in the same outpatient setting. A high dose of ivermectin (400–600 µg/kg/d) for 3 days did not show a significant benefit for the prevention of SARS-CoV-2 infection. Similarly, early treatment with the same dose and duration of ivermectin did not reduce disease progression or hospitalization in patients with mild-to-moderate COVID-19 compared with the placebo group. No serious AEs were reported in this study. However, eye-related symptoms, particularly blurred vision, occurred more frequently in the ivermectin group, especially in those who concomitantly received favipiravir.

In this study, there was a low rate of acquiring SARS-CoV-2 infection (5%) even though the study was conducted among people with high-risk exposure. Ivermectin did not show a benefit for preventing SARS-CoV-2 infection, which is in contrast with some previous studies. A recent open-labeled randomized study evaluated 303 asymptomatic household contacts in Egypt found that the proportion of clinically diagnosed SARS-CoV-2 infections was 7.4% in the ivermectin group and 58.5% in the control group [13]. Another matched case–control study conducted in India among 186 healthcare workers who received two doses of 300 µg/kg ivermectin 3 days apart found a 73% reduction in SARS-CoV-2 infection in the following month [11]. However, these previous studies were non-randomized studies with subjective outcome measurement. The low rate of a positive RT-PCR within 14 days in our study could have several explanations. First, our study was conducted after several months of a national COVID-19 vaccination campaign; therefore, 85% of participants had already received ≥1 dose of COVID-19 vaccine. Second, all confirmed COVID-19 cases in Thailand were requested to self-quarantine at home or in designated facilities to prevent further transmission [14]. This might have resulted in the low COVID-19 incidence rates in the study.

Our study demonstrated that early treatment with ivermectin did not reduce COVID-19 disease progression or the hospitalization rate and did not increase symptom resolution.

Several randomized controlled trials on the efficacy of ivermectin for treating COVID-19 have shown conflicting results in terms of virological and clinical outcomes [15,16]. However, our study results were in line with several well-controlled studies. The study conducted in Colombia did not find any clinical benefit of a 10-day ivermectin therapy among mild-to-moderate COVID-19 cases [17]. The IVERCOR-COVID19 study did not find any benefit of ivermectin therapy on preventing hospitalization [18], and a recent study in Brazil evaluating the efficacy of 3-day ivermectin for mild-to-moderate COVID-19 with risk factors also did not reduce the rate of hospitalization within 28 days compared with placebo (14.7% vs. 16.3%, respectively) [19]. The results of studies investigating ivermectin as a COVID-19 treatment may depend on the study quality [20].

The lack of observed differences in clinical outcomes between ivermectin and placebo in our treatment study should not be related with using favipiravir as standard of care. From recent systematic reviews, favipiravir did not show a significant benefit on the viral clearance and mortality [21]. In addition, it is possible that our study population had a low rate of outcomes because only one-third of the participants had a co-morbidity, which may have resulted in a lower rate of disease progression.

An adequate and safe dose of ivermectin for treating COVID-19 has not been clearly established. Ivermectin’s IC_50_ against SARS-CoV-2 was found to be 2 µM, which is >35 times higher than the maximal plasma concentration after oral ivermectin administration at the approved dose of 200 µg/kg [4]. The present study used a higher daily dose (400–600 µg/kg/d) than the standard regimen, aiming to achieve a high drug concentration during peak viremia; this dosage was found to be safe and well tolerated in a previous study [22]. However, the previous pharmacokinetic (PK) study showed that an ivermectin dosage of 10 times higher than the approved dose was not sufficient to reach the required IC50 in the lungs [23]. A recent study using a high dose of ivermectin (600 µg/kg/d) for 5 days did not reduce the SARS-CoV-2 viral load [24]. Our study did not show any benefit in clinical endpoints from high-dose ivermectin (400–600 µg/kg/d for 3 days), which is in line with these previous PK and clinical studies.

The significantly higher rate of transient blurred vision in the ivermectin group has been documented. The previous malaria study reported a significant high rate of transient visual disturbance: 8% among those who receive a moderate dose of ivermectin (300 µg/kg for 3 days) and 22% among those who received a high dose of ivermectin (600 µg/kg ivermectin for 3 days) [22]. The transient visual disturbance was possibly due to ivermectin potentiating GABA release and binding, resulting in central nervous system AEs such as mydriasis. Importantly, ocular adverse events were significantly more prevalent if co-administration with favipiravir. Nevertheless, further investigation is required to confirm the possibility of ivermectin–favipiravir drug interaction and ocular AEs. It is unclear why there was a lower rate of headache in the ivermectin group. This might have occurred by chance.

Our study has some limitations. In the ivermectin prevention study, we used self-conducted rapid antigen testing to determine the presence of SARS-CoV-2, and only those with a positive rapid antigen test underwent confirmation testing by RT-PCR. However, participants using NP swab sampling for their self-conducted test could have obtained a false-negative rapid antigen result because of improper collection technique or test performance. However, a distribution of this phenomenon should have occurred similarly in both groups. In addition, the incidence of COVID-19 infection in the prevention study was much lower than we expected. This might be due to several factors, such as the majority of participants in our study have received at least one dose of COVID-19 vaccination, or the changes in the SARSCoV-2 strain from time to time. To detect the difference of the small effect in the prevention study between ivermectin and placebo, more than 3200 participants may be required. Therefore, the result of this prevention study warrants further, larger research.

## 5. Conclusions

In this double-blinded, pragmatic randomized placebo-controlled trial, ivermectin did not demonstrate a protective effect for preventing SARS-CoV-2 infection. The results also showed that ivermectin had no COVID-19 therapeutic effect in combination with standard of care (favipiravir). Transient blurred vision was significantly more common in participants who received ivermectin plus favipiravir. Therefore, ivermectin should not be used for preventing SARS-CoV-2 infection or for treating mild-to-moderate COVID-19.

## Figures and Tables

**Table 1:** Baseline characteristics of participants with a negative RT-PCR at enrollment in the ivermectin prevention study.

Characteristics	Total	Ivermectin	Placebo	*p* Value
	*n* = 536	*n* = 259	*n* = 277	
Age (years)				
Mean (SD)	37.6 (12.0)	37.8 (12.6)	37.4 (11.6)	0.727
Median (range)	37 (18, 72)	37 (18, 72)	37 (18, 60)	0.960
Gender, *n* (%)				0.930
Male	226 (42.2)	110 (42.2)	116 (42.0)	
Female	310 (57.8)	149 (57.5)	161 (58.1)	
Body weight, kg				
Median (range)	65.1 (35.3, 142.5)	64.4 (35.3, 142.5)	65.3 (37.8, 110.2)	0.995
Mean (SD)	67.0 (15.9)	67.3 (16.9)	66.7 (14.9)	0.672
≤90 kg	487 (90.9)	232 (89.6)	255 (92.1)	0.369
>90 kg	49 (9.1)	27 (10.4)	22 (7.9)	
Presence of underlying diseases, *n* (%)	158 (29.5)	73 (28.2)	85 (30.7)	0.570
Hypertension	47 (8.8)	20 (7.7)	27 (9.7)	0.447
Diabetes mellitus	25 (4.7)	10 (3.9)	15 (5.4)	0.420
Dyslipidemia	25 (4.7)	10 (3.9)	15 (5.4)	0.420
Coronary artery disease	6 (1.1)	5 (1.9)	1 (0.4)	0.112
Chronic lung diseases	1 (0.2)	0 (0.0)	1 (0.4)	1.000
Cerebrovascular disease	2 (0.4)	0 (0.0)	2 (0.7)	0.500
Cancer	7 (1.3)	3 (1.2)	4 (1.4)	1.000
Others	97 (18.1)	45 (17.4)	52 (18.8)	0.737
Duration between last exposure to a COVID-19 patient and enrollment (*n* = 495)				1.000
Median (range)	2 (0, 66)	2 (0, 17)	3 (0, 66)	0.336
≤7 days	443 (89.5)	219 (89.4)	224 (89.6)	1.000
>7 days	52 (10.5)	26 (10.6)	26 (10.4)	
Exposure risk: household contact	495 (92.4)	245 (94.6)	250 (90.3)	0.073
Presence of symptoms, *n* (%)				
Asymptomatic	206 (38.4)	104 (40.2)	102 (36.8)	0.477
Symptomatic	330 (61.6)	155 (59.8)	175 (63.2)	
Sore throat	186 (34.7)	91 (35.1)	95 (34.3)	0.856
Cough	136 (25.4)	57 (22.0)	79 (28.5)	0.092
Runny nose	89 (16.6)	43 (16.6)	46 (16.6)	1.000
Fever	73 (13.6)	32 (12.4)	41 (14.8)	0.451
Dyspnea	27 (5.0)	9 (3.5)	18 (6.5)	0.118
Diarrhea	19 (3.5)	9 (3.5)	10 (3.6)	1.000
Chest pain	6 (1.1)	2 (0.8)	4 (1.4)	0.687
Vomiting	5 (0.9)	4 (1.5)	1 (0.4)	0.202
Loss of taste/smell	4 (0.7)	0 (0.0)	4 (1.4)	0.124
Others	114 (21.3)	52 (20.1)	62 (22.4)	0.528
Duration of illness, (*n* = 330)				
Median (range)	2 (0, 20)	2 (0, 20)	2 (0, 14)	0.692
<3 days	191 (57.9)	88 (56.8)	103 (58.9)	0.738
≥3 days	139 (42.1)	67 (43.2)	72 (41.1)	
Previous COVID-19 vaccination, *n* (%)				0.604
No	85 (15.9)	45 (17.4)	40 (14.4)	
Incomplete vaccine course (1 dose with last dose < 2 weeks prior)	34 (6.3)	13 (5.0)	21 (7.6)	
Incomplete vaccine course (1 dose with last dose ≥ 2 weeks prior)	185 (34.5)	92 (35.5)	93 (33.6)	
Completed vaccine course (2 doses with last dose < 2 weeks prior)	64 (11.9)	32 (12.4)	32 (11.6)	
Completed vaccine course (2 doses with last dose ≥ 2 weeks prior or 3 doses with any duration)	168 (31.3)	77 (29.7)	91 (32.9)	
Compliance with study medication				0.884
Full compliance, *n* (%)	485 (90.5)	235 (90.7)	250 (90.3)	
Partial compliance, *n* (%)	51 (9.5)	24 (9.3)	27 (9.7)	

SD: standard deviation.

**Table 2:** Primary outcomes of the ivermectin prevention study classified by ITT and mITT analyses.

Primary Outcomes	Ivermectin	Placebo	*p* Value
ITT analysis (*n* = 536)	*n* = 259	*n* = 277	
Proportion of COVID-19 infection within 14 days, *n* (%)	18 (6.95)	19 (6.86)	1.000
Difference (95% CI)	0.09% (−4.30–4.57)		
Median (range) time to positive SARS-CoV-2 test (days)	6 (3, 11)	6 (1, 14)	0.327
Modified ITT analysis (*n* = 525)	*n* = 253	*n* = 272	
Proportion of COVID-19 infection within 14 days, *n* (%)	12 (4.74)	14 (5.15)	0.844
Difference (95% CI)	−0.41% (−4.28–3.53)		
Median (range) time to positive SARS-CoV-2 test (days)	6 (3, 11)	4.5 (1, 14)	0.374
Ct value of participants who became positive within 14 days, mean (SD) *			
N gene	18.0 (2.8)	16.8 (3.0)	0.418
E gene	14.3 (2.9)	13.3 (3.0)	0.456
RdRp gene	18.9 (2.8)	18.1 (2.8)	0.674

* The Ct data were available for only 22 participants (10 in ivermectin group and 12 in placebo group). Four participants who became RT-PCR positive were tested at another hospital.

**Table 3:** Baseline characteristics of participants with a positive RT-PCR at enrollment in the ivermectin treatment study.

Characteristics	Total	Ivermectin	Placebo	*p* Value
	*n* = 447	*n* = 233	*n* = 214	
Age (years)				
Mean (SD)	39.5 (12.1)	39.1 (12.0)	39.8 (12.3)	0.570
Median (range)	39 (18, 72)	39 (18, 69)	40 (18, 72)	0.612
Gender, *n* (%)				0.566
Male	193 (43.2)	104 (44.6)	89 (41.6)	
Female	254 (56.8)	129 (55.4)	125 (58.4)	
Body weight, kg				
Median (range)	66.2 (36.3, 138.0)	66.3 (36.3, 138.0)	66.2 (36.6, 118.5)	0.598
Mean (SD)	68.5 (16.1)	68.1 (16.3)	69.0 (15.9)	0.608
≤90 kg, *n* (%)	406 (90.8)	214 (91.8)	192 (89.7)	0.512
>90 kg, *n* (%)	41 (9.2)	19 (8.2)	22 (10.3)	
Presence of underlying diseases, *n* (%)	143 (32.0)	70 (30.0)	73 (34.1)	0.363
Hypertension	50 (11.2)	22 (9.4)	28 (13.1)	0.233
Diabetes mellitus	31 (6.9)	14 (6.0)	17 (7.9)	0.460
Dyslipidemia	25 (5.6)	12 (5.2)	13 (6.1)	0.686
Coronary artery disease	8 (1.8)	4 (1.7)	4 (1.9)	1.000
Chronic kidney disease	2 (0.4)	1 (0.4)	1 (0.5)	1.000
Cirrhosis	1 (0.2)	1 (0.4)	0 (0.0)	1.000
Chronic lung diseases	1 (0.2)	0 (0.0)	1 (0.5)	0.481
Cerebrovascular disease	1 (0.2)	0 (0.0)	1 (0.5)	0.481
Cancer	1 (0.2)	0 (0.0)	1 (0.5)	0.481
Autoimmune disease	2 (0.4)	0 (0.0)	2 (0.9)	0.229
Others	62 (13.9)	36 (15.5)	26 (12.1)	0.340
Exposure risk: household contact, *n* (%)	314 (70.2)	158 (67.8)	156 (72.9)	0.256
Duration between last exposure to a COVID-19 patient and enrollment (*n* = 313)				
Median (range)	2 (0, 25)	2.5 (0, 25)	2 (0, 16)	0.356
≤7 days, *n* (%)	292 (93.3)	146 (92.4)	146 (94.2)	0.653
>7 days, *n* (%)	21 (6.7)	12 (7.6)	9 (5.8)	
Presence of symptoms, *n* (%)				
Asymptomatic	52 (11.6)	24 (10.3)	28 (13.1)	0.379
Symptomatic	395 (88.4)	209 (89.7)	186 (86.9)	
Cough	226 (50.6)	129 (55.4)	97 (45.3)	0.037
Sore throat	210 (47.0)	115 (49.4)	95 (44.4)	0.299
Fever	170 (38.0)	90 (38.6)	80 (37.4)	0.845
Runny nose	156 (34.9)	85 (36.5)	71 (33.2)	0.488
Loss of taste/smell	79 (17.7)	34 (14.6)	45 (21.0)	0.083
Dyspnea	31 (6.9)	21 (9.0)	10 (4.7)	0.093
Diarrhea	25 (5.6)	12 (5.2)	13 (6.1)	0.686
Chest pain	5 (1.1)	2 (0.9)	3 (1.4)	0.674
Vomiting	2 (0.4)	1 (0.4)	1 (0.5)	1.000
Others	126 (28.2)	70 (30.0)	56 (26.2)	0.400
Duration of illness, (*n* = 394)				
Median (range)	2 (0, 10)	2 (0, 10)	2 (0, 10)	0.990
<3 days, *n* (%)	219 (55.6)	115 (55.3)	104 (55.9)	0.919
≥3 days, *n* (%)	175 (44.4)	93 (44.7)	82 (44.1)	
RT-PCR Ct value				
Mean (SD)	20.2 (5.3)	20.0 (5.2)	20.4 (5.4)	0.460
<20, *n* (%)	266 (59.5)	141 (60.5)	125 (58.4)	0.700
≥20, *n* (%)	181 (40.5)	92 (39.5)	89 (41.6)	
Oxygen saturation (%), mean (SD)	97.9 (1.1)	97.9 (1.0)	97.9 (1.2)	0.964
Oxygen saturation < 96%, *n* (%)	6 (1.3)	1 (0.4)	5 (2.3)	0.109
WHO clinical score, median (range)	2 (1, 2)	2 (1, 2)	2 (1, 2)	0.360
Score 1, *n* (%)	52 (11.6)	24 (10.3)	28 (13.1)	0.379
Score 2, *n* (%)	395 (88.4)	209 (89.7)	186 (86.9)	
Previous vaccination, *n* (%)				0.522
No	112 (25.1)	65 (27.9)	47 (22.0)	
Incomplete vaccine course (1 dose with last dose < 2 weeks prior)	30 (6.7)	17 (7.3)	13 (6.1)	
Incomplete vaccine course (1 dose with last dose ≥ 2 weeks prior)	184 (41.2)	90 (38.6)	94 (43.9)	
Completed vaccine course (2 doses with last dose < 2 weeks prior)	25 (5.6)	11 (4.7)	14 (6.5)	
Completed vaccine course (2 doses with last dose ≥ 2 weeks prior or 3 doses with any duration)	96 (21.5)	50 (21.5)	46 (21.5)	
Chest X-ray, *n* (%)				0.993
Normal	264 (59.1)	138 (59.2)	126 (58.9)	
Unilateral infiltrate	7 (1.6)	4 (1.7)	3 (1.4)	
Bilateral infiltrate	6 (1.3)	3 (1.3)	3 (1.4)	
Not done	170 (38.0)	88 (37.8)	82 (38.3)	
Admission type at baseline, *n* (%)				0.072
Quarantine hotel	280 (62.6)	145 (62.2)	135 (63.1)	
Home isolation	132 (29.5)	67 (28.8)	65 (30.4)	
Hospital	33 (7.4)	21 (9.0)	12 (5.6)	
No admission	1 (0.2)	0 (0.0)	1 (0.5)	
Unknown	1 (0.2)	0 (0.0)	1 (0.5)	
Concomitant medication, *n* (%)				
Favipiravir	435 (97.5)	226 (97.4)	209 (97.7)	1.000
Others	3 (0.7)	0 (0.0)	3 (1.4)	0.110
Compliance with study medication, *n* (%)				0.762
Full compliance	399 (89.3)	209 (89.7)	190 (88.8)	
Partial compliance	48 (10.7)	24 (10.3)	24 (11.2)	

Ct: cycle threshold; RT-PCR: reverse transcription-polymerase chain reaction; SD: standard deviation; WHO: World Health Organization.

**Table 4:** Primary outcomes of the ivermectin treatment study classified by ITT and mITT analyses.

Primary Outcomes	Ivermectin	Placebo	*p* Value
ITT analysis (*n* = 447)	*n* = 233	*n* = 214	
Proportion of participants with oxygen desaturation, *n* (%) **			
Day 3	2 (0.9)	3 (1.4)	0.674
Day 7	2 (0.9)	4 (1.9)	0.433
Day 14	6 (2.6)	4 (1.9)	0.753
Change in WHO progression score from baseline			
Day 3	0 (−3, 0)	0 (−5, 0)	0.462
Day 7	0 (−4, 0)	0 (−5, 0)	0.256
Day 14	1 (−5, 1)	1 (−5, 1)	0.348
Absence of all symptoms, *n* (%)			
Day 3	57 (24.5)	44 (20.6)	0.365
Day 7	118 (50.6)	115 (53.7)	0.570
Day 14	174 (74.7)	176 (82.2)	0.066
Hospitalization due to clinical progression within 14 days, *n* (%)	8 (3.4)	4 (1.9)	0.386
28-day mortality	0	0	-
Modified ITT analysis (*n* = 443)	*n* = 229	*n* = 214	
Proportion of participants with oxygen desaturation, *n* (%) **			
Day 3	2 (0.9)	3 (1.4)	0.676
Day 7	2 (0.9)	4 (1.9)	0.435
Day 14	6 (2.7)	4 (1.9)	0.752
Change in WHO progression score from baseline			
Day 3	0 (−3, 0)	0 (−5, 0)	0.436
Day 7	0 (−4, 0)	0 (−5, 0)	0.239
Day 14	1 (−5, 1)	1 (−5, 1)	0.501
Absence of all symptoms, *n* (%)			
Day 3	56 (24.5)	44 (20.6)	0.364
Day 7	118 (51.5)	115 (53.7)	0.703
Day 14	174 (76.0)	176 (82.2)	0.129
Hospitalization due to clinical progression within 14 days, *n* (%)	4 (1.7)	4 (1.9)	1.000
28-day mortality	0	0	-

** Oxygen desaturation refers to oxygen saturation < 96% or a decrease in oxygen saturation ≥ 3% after exertion; CI: confidence interval; Ct: cycle threshold; ITT: intention to treat; SD: standard deviation; WHO: World Health Organization.

**Table 5:** Adverse events reported by all participants in the ivermectin prevention and treatment studies.

AEs (mITT Population)	Ivermectin (*n* = 482)		Placebo (*n* = 486)		*p* Value
	No. Events	No. Cases *n* (%)	No. Events	No. Cases *n* (%)	
Total	141	104 (21.6)	144	92 (18.9)	0.337
Ocular problems	28	27 (5.6)	4	3 (0.6)	<0.001
Diarrhea	23	23 (4.8)	21	19 (3.9)	0.532
Myalgia	15	13 (2.7)	19	17 (3.5)	0.579
Headache	10	9 (1.9)	25	22 (4.5)	0.027
Neurologic symptoms	8	8 (1.7)	11	10 (2.1)	0.813
Rash	7	7 (1.5)	4	4 (0.8)	0.383
Nausea/vomiting	6	6 (1.2)	12	11 (2.3)	0.328
Pruritus	1	1 (0.2)	3	3 (0.6)	0.624
Others	43	40 (8.3)	45	44 (9.1)	0.732

AE: adverse event; mITT: modified intention to treat.

## Data Availability

The datasets generated and/or analyzed during the current study are available from the corresponding author on reasonable request.

## Supplementary Materials

The following supporting information can be downloaded at: https://www.mdpi.com/article/10.3390/antibiotics11060796/s1, Supplementary File S1: Study protocol Ivermectin COVID; Figure S1: Details of symptoms by day of follow-up among participants with COVID-19 in the ivermectin treatment study; Table S1: Baseline characteristics of all study participants; Table S2: Primary outcomes in subgroup population of ivermectin prevention study by analysis of mITT population; Table S3: Primary outcomes in subgroup population of ivermectin treatment study by analysis of mITT population; Table S4: Factors associated with favorable outcome at Day 7 in treatment study; Table S5: Adverse events of study medication separated by prevention or treatment study. References [4,12,18,25,26,27,28,29,30,31] are cited in the supplementary materials.
