# Supplementary material for: Safety and Efficacy of Ivermectin for the Prevention and Treatment of COVID-19: A Double-Blinded Randomized Placebo-Controlled Study
Source: Antibiotics (Basel). 2022 Jun 12;11(6):796. doi: 10.3390/antibiotics11060796 (PMC9219629; doi:10.3390/antibiotics11060796)
Supplement: Supplementary file 1 [file antibiotics-11-00796-s001.zip › Study protocol Ivermectin COVID.pdf]

Study protocol

Safety and Efficacy of ivermectin for the prevention and treatment of COVID-19: a double-blinded randomized placebo-controlled study

Version 1.0, July 2021

Contents

Sections	Page
1. Study background	3
2. Objectives	5
3. Methodology	
• Study design	6
• Study site	6
• Sample size	6
• Inclusion criteria	7
• Exclusion criteria	7
• Withdrawal criteria	7
• Study schedule and procedures	7
• Study medication	13
• Outcome measurement	13
• Safety monitoring	14
• Statistical analysis plan	14
4. Ethical issues	16
5. Case record form	17
6. Reference	20
7. Principle investigator contact	21

Table index

Description	Page
Table 1: Concomitant medications which are not allowed in the study	7
Table 2: the study activity	11
Table 3: WHO clinical progression scales	11
Table 4: number of drugs adjusted by patient body weight.	13

Figure index

Description	Page
Figure 1: study scheme	12

1. Study background

There has been an outbreak of newly emerged coronavirus disease so-called COVID-19 around the world. In Thailand, the cumulative case of SARs-CoV-2 infected patients has reached 283,067 by July 2021 with 2,226 death (0.8%). The outbreak was robust during April to January 2021 caused by the predominance of Delta SARs-CoV-2 variant. The published data from the ministry of health showed that there were 246,576 patients infected with SARs-CoV-2 during April 1st 2021 to July 3rd 2021[1].

Most COVID-19 patients presented with upper respiratory tract symptoms such as rhinorrhea, sore throat, fever and cough. However, pneumonia was noticed in approximate 20-30%, 5% of which require intensive care monitoring and ventilator support[2]. The effective antiviral treatments for COVID-19 are still under research and development. In Thailand, favipiravir is recommended in patients with mild to moderate symptomatic COVID-19 or asymptomatic patients with risks for severe disease progression, including those with age >60 years or <5 years, chronic pulmonary disease, chronic kidney disease, cardiovascular disease, cerebrovascular disease, hypertension, diabetes mellitus, obesity (body mass index ≥35 kg/m^2), liver cirrhosis, immunocompromised status, and/or lymphocyte count <1000 cells/mm[3]. Intravenous remdesivir is given for patients with more severe diseases such as patients who require oxygen therapy. A corticosteroid for 7-10 days is also given in patients who have COVID-19 pneumonia (1).

Currently, *in vitro* studies show that ivermectin, which is an anti-parasitic drug approved for the treatment of strongyloidiasis, exhibited activity against SARs-CoV-2. It can inhibit nuclear translocation of SARs-CoV-2[3]. Viral replication could be decreased by 5000 times within 48 hours after ivermectin commencement. The IC 50 is 2.5 micromole and drug concentration of 2-4 microgram/ml is needed to achieve adequate IC50. However, the required dosage is higher than usual therapeutic dosage (200 mcg/kg) used for parasitic treatment by 50-100 times. Nevertheless, safety profile of ivermectin has been demonstrated in healthy volunteer who received high-dose ivermectin (10 times of usual doses)[4]. Various studies showed that ivermectin demonstrated the efficacy in viral replication inhibition, and the drug could be used with other antimicrobial agents such as

hydroxychloroquine, doxycycline and azithromycin[5]. Ivermectin is affordable and widely available. Moreover, ivermectin has an anti- inflammatory effect by inhibition of nitric oxide, prostaglandin and interleukin-6.

As aforementioned result, ivermectin has been used widely in many countries especially in South Asia such as India. However, recent studies showed conflicting results. The systematic review included 10 randomized controlled trials which enrolled 1,173 patients with COVID-19. Most participants were mild to moderate disease severity. The dosage of ivermectin ranged from 12 to 210 mg/course of treatment, and the duration varied from 1-5 days. The results showed that treatment with ivermectin resulted in trend towards reduced mortality rate (RR 0.37; 95%CI 0.12-1.13) when compared with standard treatment. The adverse events were not statistically significant different (RR 0.95; 95% CI 0.85-1.07). The viral clearance also did not differ (RR 0.96; 95%CI 0.79-1.16) [6].

Another systematic review included 24 randomized control trials which enrolled 3,328 COVID-19 patients. The dosage of ivermectin ranged from 200-600 mcg/kg/day for 5 days. Most patients were also mild to moderate disease severity. Interestingly, the result showed that ivermectin could reduce inflammation and promote viral clearance especially when used at the higher dosage. The mortality was also decreased by 56% [7].

There are still ongoing studies on effectiveness of ivermectin in COVID-19 treatment such as IVERCOR-COVID-19. The study is a randomized controlled trial in Argentina which is aimed to include 500 participants. The ivermectin of 2-4 tablets/day for 2 days were given in this study and the primary outcome was hospitalization rate [8].

Many studies from African countries which used ivermectin as mass drug administration (MDA) for parasitic eradication revealed lower incidence of COVID-19 [9]. All data support the off-labeled use of ivermectin in COVID-19. However, most studies focused only on the efficacy on treatment of COVID-19. The sample sizes are limitation of most studies. Moreover, the study of ivermectin as COVID-19 prevention are very scarce.

Therefore, we perform this study to investigate the efficacy of ivermectin in prevention and treatment of COVID-19.

2. Objectives

2.1 Primary objectives

- To study the efficacy of ivermectin in prevention of COVID-19 compared with placebo
- To study the efficacy of ivermectin plus standard of care in treatment of mild to moderate COVID-19 compared with placebo plus standard of care

2.2 Secondary objectives

- To determine the safety of the study medication

3. Methodology

3.1 Study design

This study is a double-blinded, pragmatic, randomized placebo-controlled trial.

3.2 Study site

This is a single-center study. Participants will be recruited from patients who visit an acute respiratory tract infection (ARI) outpatient clinic at a university-based 2,300-bed referral center in Bangkok, Thailand.

3.3 Sample size

For the prevention study, the true proportion of participants with a negative RT-PCR SARS-CoV-2 test who subsequently had a positive RT-PCR SARS-CoV-2 test among the high-risk patients is unknown. Therefore, it is assumed that a 3-day course of ivermectin would reduce the rate of SARS-CoV-2 infection from 20% to 10%. To achieve a power of 80% and a two-sided significance level of 0.05, 199 participants with a negative RT-PCR SARS-CoV-2 test at enrollment were required in each group. We expect that 20% of participants will have incomplete study information or would be lost to follow-up; thus, 478 participants with a negative RT-PCR SARS-CoV-2 test at enrollment are needed.

For the treatment study, it is assumed that ivermectin would reduce the rate of oxygen desaturation of COVID-19 patients from 30% to 15%. To achieve 80% power and a two-sided significance level of 0.05, 121 patients with a positive RT-PCR SARS-CoV-2 test at enrollment were required in each group. We expect that 20% of participants would have incomplete study information or be lost to follow-up; therefore, 290 patients with a positive RT-PCR SARS-CoV-2 test at enrollment were needed.

Because there is approximately 50% prevalence of SARS-CoV-2 infection among the patients who visited the ARI clinic, we intend to enroll at least 1,000 patients who presented to the ARI clinic to achieve the target sample size for both the ivermectin prevention and treatment studies.

3.4 Inclusion criteria

1. Participants at age 18-year-old or older
2. Having performed NP swab at ARI clinic due to acute respiratory tract symptoms with/or without fever, or a history of contact confirmed cases of COVID-19 with documented positive or negative RT-PCR.
3. Be able and fluent to communicate via Thai language
4. Be able to be contact via telephone call during the study
5. Be willing to give signed inform consent before the study enrollment

3.5 Exclusion criteria

1. History of confirmed COVID-19 within 90 days of study enrollment
2. Suspected or confirmed pregnancy or breastfeeding women
3. History of ivermectin hypersensitivity
4. Receive concomitant medications which could have significant drug interaction with ivermectin as the following table **(Table 1)**
5. RT-PCR demonstrate inconclusive result

Table 1: Concomitant medications which are not allowed in the study

Drug names			
Barbiturates	Benzodiazepines	Sodium oxybutyrate	Valproic acid
Amiodarone	Carvedilol	Verapamil	Clarithromycin
Erythromycin	Itraconazole	Ketoconazole	Rifampicin
Ritonavir	Quinidine	Tamoxifen	Phenothiazines
Cyclosporin			

3.6 Withdrawal criteria

1. Participants are willing to withdraw the informed consent
2. Serious adverse drug reaction

3.7 Study schedules and procedures

3.7.1. Participant screening

Participants who are interested in the study will receive the information. They will be screened on inclusion and exclusion criteria. All questions will be answered.

3.7.2. Participant enrollment and informed consent

To reduce likelihood of SARS-CoV-2 contamination during participant enrollment, informed consent will be made via electronic signed in electronic tablets. Informed consent must be obtained prior to participant enrollment.

3.7.3. At the enrollment date (D0)

The study investigator will take the patient history, record body weight, vital sign and oxygen saturation. The contact history and respiratory symptoms will be obtained in detail. Baseline characteristics, comorbidities and concurrent medications are also recorded.

Participants are randomized in a 1:1 ratio to receive study drug (ivermectin) or placebo (please see section 3.8 for detail). The randomization is done by the study's unmasked data team using computer-generated random numbers with a randomly selected block size between 2 and 8.

The pharmacist assigns the letter A or B to each participant and prepared visually matched pills in identical pre-packed plastic bags and sequentially numbered the treatment packs according to the randomization lists. The letter (A or B) and randomized number will be labeled on the ivermectin or placebo bags, and the tablet bags were dispensed in sequential order as participants were recruited. Only the pharmacist knows which treatment letter indicated ivermectin or placebo. The pharmacist is not involved in any of the subsequent trial procedures. The participants and investigators are blinded to the treatment assignment and remained unaware of the assigned treatment until all participants had undergone their 28-day follow-up visit. The patient will receive NP and throat swab for SARS-CoV-2 PCR according to the hospital protocol. The result of PCR will be reported within 24 hours.

3.7.4 After the enrollment date

After the SARS-CoV-2 RT-PCR result available, which is usually on the enrollment day, the participants who are negative for SARS-

CoV-2 will be included in the ivermectin prevention study, and those who are positive for SARS-CoV-2 will be included in the ivermectin treatment study. Therefore, the participants will be allocated to prevention or treatment arm based on PCR results.

COVID-19 uninfected patients: The participants in this group are instructed to collect NP swab for the rapid detection of SARS-CoV-2 antigen using standard Q COVID-19 antigen test (SD Biosensor, Inc, Gyeonggi-do, Korea) if they develop new symptoms suggestive of COVID-19 or at day 14 if they are asymptomatic. In case of positive rapid antigen test, RT-PCR will be performed at the hospital.

COVID-19 infected patients: The participants in this group will receive standard treatment and will be treated at home (home isolation), or designated facility (quarantine hotel or hospital) by the primary physicians. Antiviral medication either favipiravir or remdesivir are allowed in this study and can be given as appropriate. Participants in the treatment study are instructed to measure their temperature and oxygen saturation on days 3, 7, and 14, and will be asked about the current treatment location (quarantine hotel, home or hospital) and the type of oxygen supplement (if required) at each time point.

All participants will be contacted by telephone on days 3, 7, and 14 to assess the safety of the study medication, the compliance rate, and clinical status. They will be asked whether they had experienced symptoms of possible adverse events (AEs), including headache, pruritus, rash, myalgia, nausea, vomiting, diarrhea, ocular problems, or neurological and hematological AEs. The presence of new symptoms and the absence of previous symptoms at each time point are also collected. The list of symptoms that are assessed included fever, runny nose, cough, sore throat, chest pain, dyspnea, diarrhea, vomiting, and loss of taste and/or smell. **Table 2** shows the schedule of research activity performed in this study. Ten-point clinical progression scale endorsed by WHO is used in this study (**Table 3**).

Table 2: The study schedule

Activity	D0 (enrollment)	D3	D7	D14	D28
History taking	☑				
Physical examination	☑				
NP swab for RT-PCR	☑				
Drug dispensing	☑				
Telephone call					
- Clinical assessment		☑	☑	☑	☑
- Drug adherence		☑	☑	☑	
- Safety assessment		☑	☑	☑	
NP swab (prophylaxis arm)		☑ (if new symptoms)		☑	

Table 3: WHO clinical progression scales [10]

Patient status	Descriptor	Score
Uninfected	PCR for SARS-CoV-2 negative	0
Ambulatory, mild disease	Asymptomatic patients	1
	Symptomatic: independent	2
	Symptomatic: assistance needed	3
Hospitalized, moderate disease	Hospitalized, no oxygen therapy	4
	Hospitalized, oxygen cannula or mask	5
Hospitalized, severe disease	Hospitalized, oxygen by NIV or high flow	6
	Intubation and mechanical ventilation, $pO_2/FiO_2 \geq 150$	7
	Intubation and mechanical ventilation, $pO_2/FiO_2 < 150$ or vasopressors	8
	Intubation and mechanical ventilation, $pO_2/FiO_2 \geq 150$ and vasopressors, dialysis or ECMO	9

Death	Death	10

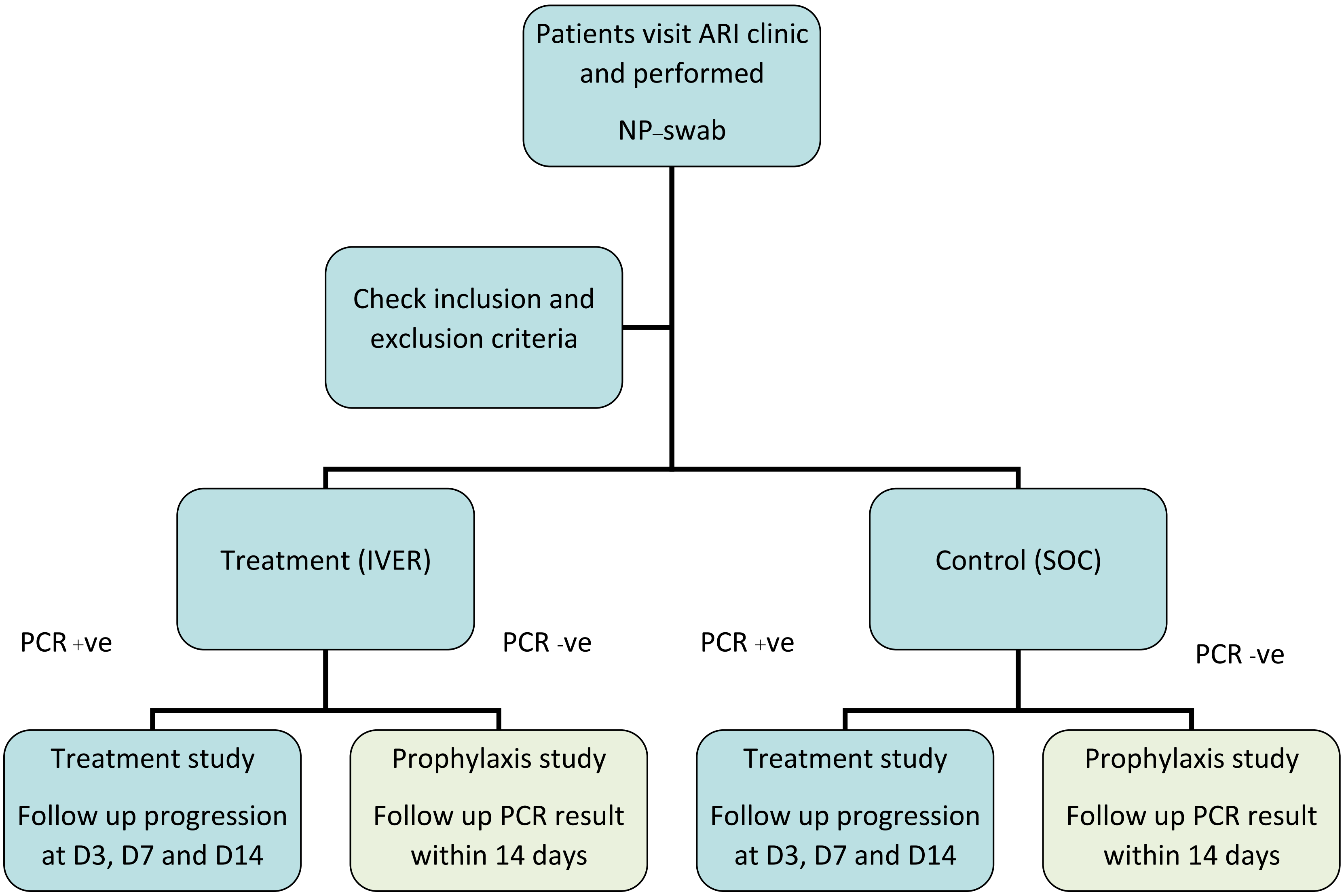

Figure 1; study scheme

3.8 Study medication

The study medication is ivermectin (Atlantic Laboratory Ltd, Bangkok, Thailand). The drug and placebo are identical in color and size. Participants are given either placebo or ivermectin based on their body weight; the ivermectin dose ranged from 400 to 600 µg/kg/d. The actual body weight is used to calculate the patient weight. The dosage is calculated to the nearest 6-mg or 12-mg whole tablets. The participants are advised to take the study medication before a meal on the enrollment day (day 0) and once every 24 h thereafter for the subsequent 2 consecutive days. The participants will receive study drug or placebo 3-6 tablets/day depending on body weights. The numbers of study medication are shown in the **Table 4.**

Table 4: Number of drugs adjusted by patient body weight.

Body weight	Drugs (tablets/day, 6 mg/tab)
30-45 kg	3
> 45-60 kg	4
> 60-75 kg	5
> 75 lg	6

3.9 Outcome measurement

The primary outcome of the prevention study is the proportion of participants with a positive RT-PCR SARS-CoV-2 test within 14 days after enrollment among those with a negative RT-PCR result at enrollment in the modified ITT population.

The primary outcomes of the treatment study are the proportion of participants with oxygen desaturation (oxygen saturation <96% or decreased from baseline by ≥3% after exertion), changes in the WHO 10-point clinical progression score [10] at days 3, 7, and 14 compared to baseline, the absence of all symptoms at days 3, 7, and 14, the hospitalization rate within 14 days, and the 28-day mortality rate in the modified ITT population.

The secondary outcome of the study is the safety of the study medications, including the number of AEs and the percentage of participants with AEs evaluated in the modified ITT population.

3.10 Safety monitoring

All participants will be contacted by telephone on days 3, 7, and 14 to assess the safety of the study medication.

They will be asked whether they have experienced symptoms of possible adverse events (AEs), including headache, pruritus, rash, myalgia, nausea, vomiting, diarrhea, ocular problems, or neurological and hematological AEs.

The presence of new symptoms and the absence of previous symptoms at each time point will also be collected.

The list of symptoms that will be assessed included fever, runny nose, cough, sore throat, chest pain, dyspnea, diarrhea, vomiting, and loss of taste and/or smell.

A Serious Adverse Event is any untoward medical occurrence at any dose that:

- Results in death or;
- Is life-threatening (at risk of death at the time of the event)
- Requires inpatient hospitalization or prolongation of existing hospitalization or;
- Results in persistent or significant disability/incapacity or;
- Is a congenital anomaly/birth defect;
- Is a medically important event:

3.11 Statistical analysis plan

Demographic information and baseline characteristics of the participants will be presented as descriptive statistics. Continuous data will be presented as the mean (standard deviation (SD)) or median (range), as appropriate. Categorical data will be presented as number (n) and percentage (%). The unpaired t-test and Mann–Whitney U test will be used to compare continuous data with normal and non-normal distributions, and the chi-square test or Fisher's exact test will be used to compare categorical data. All statistical analyses will be performed

with PASW Statistics (SPSS) 18.0 (IBM Corp., Armonk, NY, USA), which a p-value $\leq$0.05 is considered statistically significant.

The primary outcomes of the prevention and treatment studies will be analyzed using intention to treat (ITT) and modified intention to treat (mITT) populations.

The ITT population comprised all eligible participants who are randomized and applied a worst-case scenario that assumed all participants who withdraw or do not take the study drug, and those in the prevention study who do not perform the second NP swab, have a poor outcome. The mITT population includes all randomized participants who receive at least one dose of study drug. Participants in the prevention study who do not perform a second NP swab within 14 days are assumed to have a negative RT-PCR result in the mITT population if they are asymptomatic at day 28 without proof of a RT-PCR test taken elsewhere.

Primary outcome of the prevention study will be analyzed in mITT population by the subgroup of duration of contact (≤7 days vs. >7 days), body weight (≤90 kg vs. >90 kg) and vaccination status (no immune, partially immune and fully immune) as the following defined criteria: no immune (never receive COVID-19 vaccine or received one dose of any vaccine less than 2 weeks), partially immune (received one dose of any vaccine 2 weeks or longer or two dose of any vaccine less than 2 weeks) and fully immune (received two dose of any vaccine more than 2 weeks or receive third dose booster for any duration).

Primary outcomes of the treatment study will be analyzed in mITT population by the subgroup of duration of body weight (≤90 kg vs. >90 kg), duration of illness (<3 days vs. ≥3 days), cycle threshold (<20 vs. ≥20), concomitant favipiravir (with favipiravir vs. no favipiravir) and vaccination status (no immune, partially immune and fully immune) as defined above.

4. Ethical issues

The study protocol was reviewed and approved by the Siriraj Institutional Review Board (certificate of approval no. Si 607/2021) and was conducted in accordance with the Declaration of Helsinki.

A signed inform consent form will be obtained from the subject via electronic sign using IPAD. For subjects who cannot consent for themselves, such as those below the legal age, a parent, legal guardian, or person with power of attorney, must sign the consent form; additionally, the subject's assent must also be obtained if he or she is able to understand the nature, significance, and risks associated with the study.

The inform consent form will describe the purpose of the study, the procedures to be followed, and the risks and benefits of participation. A copy of the consent form will be given to the subject, parent, or legal guardian, and this fact will be documented in the subject’s record.

5. Case record form

Case record form: Ivermectin for COVID-19		
Study number	☐☐☐☐☐	
**Part 1: Demographic data**		
Sex	☐Male	☐ Female
Age	☐☐ years	
Body weight	☐☐ kilograms	
Underlying disease	☐ Diabetes	☐ Hypertension
	☐ Dyslipidemia	☐ CAD
	☐ CKD	☐ Cirrhosis
	☐ Chronic lung dis	☐ CVA
	☐ Cancer (pls specify) _________	
	☐ Autoimmune disease _____	
	☐ Others (pls specify) ___________	
**Part 2: Inclusion criteria checklist**		
Age >18 years old	☐ Yes	☐ No
Received NP swab for COVID-19	☐ Yes	☐ No
Not in pregnant state or breastfeeding	☐ Yes	☐ No
No history of ivermectin allergy	☐ Yes	☐ No
No GABA potentiating activity drugs	☐ Yes	☐ No
No previous history of COVID-19 within 90 days	☐ Yes	☐ No
**Part 3: Clinical data at enrollment date (D0)**		
Date of 1st NP swab	☐☐/☐☐/☐☐☐☐	
Date of first symptoms (if present)	☐☐/☐☐/☐☐☐☐	
Symptoms at date of 1st NP swab	☐ Fever	☐ Runny nose
	☐ Cough	☐ Sore throat
	☐ Chest pain	☐ Dyspnea
	☐ Diarrhea	☐ Vomiting
	☐ Loss of smell/taste	
	☐ Others ______________	
	☐ No symptoms	
Previous COVID-19 vaccination	☐ no	☐ yes, please specify

	☐ SV ☐ one dose ☐ two doses<br>Date of last dose ☐☐/☐☐/☐☐☐☐<br>☐ AZ ☐ one dose ☐ two doses<br>Date of last dose ☐☐/☐☐/☐☐☐☐
Vital signs at date of enrollment	T ☐☐ RR☐☐
	P ☐☐☐ SpO_2 ☐☐
NP swab result	☐ Positive ☐ Negative
	CT value (N gene) ☐☐
Chest x-ray at enrollment (if available)	☐ Normal
	☐ Unilateral infiltrate
	☐ Bilateral infiltrate
	☐ Not applicable
Diagnosis	☐ Asymptomatic COVID-19
	☐ COVID-19 URI
	☐ COVID-19 pneumonia
	☐ No infection
**Part 4: Intervention**	
Number of medication/day	☐ 3 ☐ 4 ☐ 5 ☐ 6
Study group	☐ Prophylaxis ☐ Treatment
**Part 5: Admission and other treatment data(for treatment group only)**	
Date of admission (if present)	☐☐/☐☐/☐☐☐☐
Type of admission	☐ Home isolation
	☐ Quarantine hotel
	☐ Hospital ________ ward ________
	☐ No admission
	☐ Not applicable/unknown
Receive favipiravir	☐ no ☐ yes, pls specify<br>Date of initiation ☐☐/☐☐/☐☐☐☐<br>Duration ☐☐ days
Other medication	☐ no ☐ yes, pls specify<br>Date of initiation☐☐/☐☐/☐☐☐☐<br>Duration ☐☐ days
**Part 6: Final outcome at 28 days after enrollment**	
Prevention group	
COVID-19 diagnosis	☐ Yes Date ☐☐/☐☐/☐☐☐☐<br>☐ No
Treatment group	

Pneumonia	☐ Yes Date ☐☐/☐☐/☐☐☐☐<br>☐ No
Low flow oxygen	☐ Yes Date ☐☐/☐☐/☐☐☐☐<br>☐ No
HFNC	☐ Yes Date ☐☐/☐☐/☐☐☐☐<br>☐ No
Intubation	☐ Yes Date ☐☐/☐☐/☐☐☐☐<br>☐ No
ICU admission	☐ Yes Date ☐☐/☐☐/☐☐☐☐<br>☐ No
28-day survival	☐ Survive ☐ Death
Cause of death (if present)	________________
Date of death (if present)	☐☐/☐☐/☐☐☐☐

6. Reference

1. https://ddc.moph.go.th/viralpneumonia/index.php
2. Sirijatuphat R, Suputtamongkol Y, Angkasekwinai N, Horthongkham N, Chayakulkeeree M, Rattanaumpawan P, et al. Epidemiology, clinical characteristics, and treatment outcomes of patients with COVID-19 at Thailand's university-based referral hospital. BMC Infect Dis. 2021;21(1):382.
3. Caly L, Druce JD, Catton MG, Jans DA, Wagstaff KM. The FDA-approved drug ivermectin inhibits the replication of SARS-CoV-2 in vitro. Antiviral Res. 2020;178:104787.
4. Guzzo CA, Furtek CI, Porras AG, Chen C, Tipping R, Clineschmidt CM, et al. Safety, tolerability, and pharmacokinetics of escalating high doses of ivermectin in healthy adult subjects. J Clin Pharmacol. 2002;42(10):1122-33.
5. Cavalcanti AB, Berwanger O, Zampieri FG. Hydroxychloroquine with or without Azithromycin in Covid-19. Reply. N Engl J Med. 2021;384(2):191.
6. Roman YM, Burela PA, Pasupuleti V, Piscoya A, Vidal JE, Hernandez AV. Ivermectin for the treatment of COVID-19: A systematic review and meta-analysis of randomized controlled trials. Clin Infect Dis. 2021.
7. Andrew Hill AA, Sabeena Ahmed, Asma Asghar,. Meta-analysis of randomized trials of ivermectin to treat SARS-CoV-2 infection. Research Square. 2021.
8. Vallejos J, Zoni R, Bangher M, Villamandos S, Bobadilla A, Plano F, et al. Ivermectin to prevent hospitalizations in patients with COVID-19 (IVERCOR-COVID19): a structured summary of a study protocol for a randomized controlled trial. Trials. 2020;21(1):965.
9. Hellwig MD, Maia A. A COVID-19 prophylaxis? Lower incidence associated with prophylactic administration of ivermectin. Int J Antimicrob Agents. 2021;57(1):106248.
10. WHO Working Group on the Clinical Characterisation and Management of COVID-19 infection. A minimal common outcome measure set for COVID-19 clinical research. Lancet Infect Dis. 2020;20(8):e192-e197.

7. Principle-investigator contact

Nasikarn Angkasekwinai, M.D.

Associate Professor
Division of Infectious Disease and Tropical Medicine, Department of Medicine, Faculty of Medicine Siriraj Hospital, Mahidol University, Bangkok, Thailand
E-mail address: nasikarn@gmail.com, nasikarn.ang@mahidol.ac.th
Telephone: 02-419-7783
