# Supplementary material for: Safety and Efficacy of Ivermectin for the Prevention and Treatment of COVID-19: A Double-Blinded Randomized Placebo-Controlled Study
Source: Antibiotics (Basel). 2022 Jun 12;11(6):796. doi: 10.3390/antibiotics11060796 (PMC9219629; doi:10.3390/antibiotics11060796)
Supplement: Supplementary file 1 [file antibiotics-11-00796-s001.zip › Supplementary Tables S1-S5_Ivermectin COVID.pdf]

Supplementary Table S1: Baseline characteristics of all study participants

	Total<br>n=983	Ivermectin<br>n =492	Placebo<br>n=491	*P* value
Age (years),				
Mean (SD)	38.4 (12.1)	38.4 (12.3)	38.4 (11.9)	0.972
Median (range)	38 (18, 72)	38 (18, 72)	38 (18, 72)	0.883
Gender, n (%)				0.606
Male	419 (42.6)	214 (43.5)	205 (41.8)	
Female	564 (57.4)	278 (56.5)	286 (58.2)	
Body weight, kg				
Mean (SD)	67.7 (16.0)	67.6 (16.6)	67.7 (15.3)	0.986
Median (range)	65.7 (35.3, 142.5)	65.7 (35.3, 142.5)	65.7 (36.6, 118.5)	0.786
≤90 kg	893 (90.8)	446 (90.7)	447 (91.0)	0.912
>90 kg	90 (9.2)	46 (9.3)	44 (9.0)	
Presence of underlying diseases, n (%)	301 (30.6)	143 (29.1)	158 (32.2)	0.300
Hypertension	97 (9.9)	42 (8.5)	55 (11.2)	0.166
Diabetes mellitus	56 (5.7)	24 (4.9)	32 (6.5)	0.275
Dyslipidemia	50 (5.1)	22 (4.5)	28 (5.7)	0.388
Coronary artery disease	14 (1.4)	9 (1.8)	5 (1.0)	0.421
Chronic kidney disease	2 (0.2)	1 (0.2)	1 (0.2)	1.000
Cirrhosis	1 (0.1)	1 (0.2)	0 (0.0)	1.000
Chronic lung diseases	2 (0.2)	0 (0.0)	2 (0.4)	0.249
Cerebrovascular disease	3 (0.3)	0 (0.0)	3 (0.6)	0.124
Cancer	8 (0.8)	3 (0.6)	5 (1.0)	0.506
Autoimmune disease	2 (0.2)	0 (0.0)	2 (0.4)	0.249
Others	159 (16.2)	81 (16.5)	78 (15.9)	0.863
Duration of last exposure to COVID-19 patient until enrollment (n=808)*				0.714
Median (range)	2 (0, 66)	2 (0, 25)	2 (0, 66)	0.844
≤ 7 days, n (%)	735 (91.0)	365 (90.6)	370 (91.4)	0.714
>7 days, n (%)	73 (9.0)	38 (9.4)	35 (8.6)	
Exposure risk: household contact, n (%)	809 (82.3)	403 (81.9)	406 (82.7)	0.802
Presence of symptom, n (%)				
Asymptomatic	258 (26.2)	128 (26.0)	130 (26.5)	0.885

Symptomatic	725 (73.8)	364 (74.0)	361 (73.5)	
Sore throat	396 (40.3)	206 (41.9)	190 (38.7)	0.330
Cough	362 (36.8)	186 (37.8)	176 (35.8)	0.552
Runny nose	245 (24.9)	128 (26.0)	117 (23.8)	0.461
Fever	243 (24.7)	122 (24.8)	121 (24.6)	1.000
Loss of smell/ taste	83 (8.4)	34 (6.9)	49 (10.0)	0.087
Dyspnea	58 (5.9)	30 (6.1)	28 (5.7)	0.892
Diarrhea	44 (4.5)	21 (4.3)	23 (4.7)	0.761
Chest pain	11 (1.1)	4 (0.8)	7 (1.4)	0.385
Vomiting	7 (0.7)	5 (1.0)	2 (0.4)	0.451
Others	240 (24.4)	122 (24.8)	118 (24.0)	0.824
Duration (days) of illness (n=725) **				
Median (range)	2 (0, 20)	2 (0, 20)	2 (0, 14)	0.746
< 3 days, n (%)	410 (56.6)	203 (55.9)	207 (57.3)	0.708
≥ 3 days, n (%)	314 (43.4)	160 (44.1)	154 (42.7)	
Positive RT-PCR, n (%)	447 (45.5)	233 (47.4)	214 (43.6)	0.249
Previous COVID-19 vaccination, n (%)				0.482
No	197 (20.0)	110 (22.4)	87 (17.7)	
Incomplete course of vaccine (1 dose with last dose < 2 weeks)	64 (6.5)	30 (6.1)	34 (6.9)	
Incomplete course of vaccine (1 dose with last dose ≥ 2 weeks)	369 (37.5)	182 (37.0)	187 (38.1)	
Complete course of vaccine (2 doses with last dose < 2 weeks	89 (9.1)	43 (8.7)	46 (9.4)	
Complete course of vaccine (2 doses with last dose ≥ 2 weeks or 3 doses with any duration)	264 (26.9)	127 (25.8)	137 (27.9)	
Compliance of study medication				0.752
Full compliance, n (%)	884 (89.9)	444 (90.2)	440 (89.6)	
Partial compliance, n (%)	99 (10.1)	48 (9.8)	51 (10.4)	

*Duration of last exposure to COVID-19 patients until enrollment, data was available in 808 participants

**Duration of illness: duration of illness prior to enrollment (only in symptomatic patients)

Supplementary Table S2: Primary outcomes in subgroup population of ivermectin prevention study by analysis of mITT population

Subgroup analysis of ivermectin prevention study	Ivermectin	Placebo	p-value
Proportion of partcipants with positive RT-PCR or rapid antigen test in subgroup population (n=525)	n=253	n=272	
By duration of last exposure to enrollment, n (%)*			
≤7 days	12/214 (5.61)	14/219 (6.39)	0.840
>7days	0/26 (0.00)	0/26 (0.00)	
By body weight (kg), n (%)			
≤90 kg	11/226 (4.87)	12/250 (4.80)	1.000
>90 kg	1/27 (3.70)	2/22 (9.09)	0.581
By vaccination status, n (%)**			
No immune	3/57 (5.26)	4/60 (6.67)	1.000
Partially immune	8/123 (6.50)	4/122 (3.28)	0.376
Fully immune	1/73 (1.37)	6/90 (6.67)	0.131

*Duration of last exposure with COVID-19 patient to enrollment was avalaible in 483 participants (92%); **Vaccination status was classified into three groups: no immune (never receive COVID-19 vaccine or received one dose of any vaccine less than 2 weeks), partially immune (received one dose of any vaccine 2 weeks or longer or two dose of any vaccine less than 2 weeks) and fully immune (received two dose of any vaccine more than 2 weeks or receive third dose booster for any duration).

Supplementary Table S3: Primary outcomes in subgroup population of ivermectin treatment study by analysis of mITT population

Subgroup analysis of ivermectin treatment study	Ivermectin	Placebo	*P* value
mITT population (n=443)	n=229	n=214	
Body weight (kg)			
BW ≤90 kg (n=402)	n=210	n=192	
Proportion of participants with oxygen desaturation, n (%)			
Day 3	2/210 (1.0)	3/192 (1.6)	0.673
Day 7	2/208 (1.0)	4/189 (2.1)	0.430
Day 14	5/206 (2.4)	3/191 (1.6)	0.725
Change of WHO progression score from baseline, n (%)			
Day 3	0 (-3, 0)	0 (-5, 0)	0.364
Day 7	0 (-4, 0)	0 (-5, 0)	0.306
Day 14	1 (-5, 1)	1 (-5, 1)	0.384
Absence of symptom, n (%)			
Day 3	47/210 (22.4)	39/192 (20.3)	0.628
Day 7	108/210 (51.4)	102/192 (53.1)	0.765
Day 14	161/210 (76.7)	161/192 (83.9)	0.080
Hospitalization due to clinical progression within 14 days, n (%)	3/210 (1.4)	3/192 (1.6)	1.000
BW >90 kg (n=41)	n=19	n=22	
Proportion of participants with oxygen desaturation, n (%)			
Day 3	0/19 (0.0)	0/22 (0.0)	0.676
Day 7	0/19 (0.0)	0/22 (0.0)	0.435

Day 14	1/18 (5.6)	1/21(4.8)	1.000
Change of WHO progression score from baseline			
Day 3	0 (-3, 0)	0 (-1, 0)	0.798
Day 7	0 (-3, 0)	0 (-4, 0)	0.557
Day 14	1 (0, 1)	1 (-2, 1)	0.740
Absence of symptom, n (%)			
Day 3	9/19 (47.4)	5/22 (22.7)	0.115
Day 7	10/19 (52.6)	13/22 (59.1)	0.758
Day 14	13/19 (68.4)	15/22 (68.2)	1.000
Hospitalization due to clinical progression within 14 days, n (%)	1/19 (5.3)	1/22 (4.5)	1.000
Duration of illness			
Duration <3 days (n=218)	n=114	n=104	
Proportion of participants with oxygen desaturation, n (%)			
Day 3	2/114 (1.8)	1/104 (1.0)	1.000
Day 7	2/113 (1.8)	1/103 (1.0)	1.000
Day 14	4/111 (3.6)	2/103 (1.9)	0.684
Change of WHO progression score from baseline, n (%)			
Day 3	0 (-3, 0)	0 (-3, 0)	0.439
Day 7	0 (-4, 0)	0 (-3, 0)	0.676
Day 14	1 (-5, 1)	1 (-2, 1)	0.258
Absence of symptom			
Day 3	25/114 (21.9)	19/104 (18.3)	0.613
Day 7	57/114 (50.0)	50/104 (48.1)	0.788

Day 14	88/114 (77.2)	86/104 (82.7)	0.399
Hospitalization due to clinical progression within 14 days, n (%)	3/114 (2.6)	2/104 (1.9)	1.000
Duration ≥3 days (n=173)	n=91	n=82	
Proportion of participants with oxygen desaturation, n (%)			
Day 3	0/91 (0.0)	2/82 (2.4)	0.223
Day 7	0/90 (0.0)	2/80 (2.5)	1.000
Day 14	2/90 (2.2)	1/82 (1.2)	1.000
Change of WHO progression score from baseline, n (%)			
Day 3	0 (-3, 0)	0 (-3, 0)	0.387
Day 7	0 (-2, 0)	0 (-4, 0)	0.109
Day 14	1 (0, 1)	1 (-2, 1)	0.374
Absence of symptom, n (%)			
Day 3	24/91 (26.4)	15/82 (18.3)	0.274
Day 7	46/91 (50.5)	46/82 (56.1)	0.542
Day 14	66/91 (72.5)	67/82 (81.7)	0.206
Hospitalization due to clinical progression within 14 days	0/91 (0.0)	2/82 (2.4)	0.223
Cycle threshold (Ct) value			
Ct <20 (n=264)	n=139	n=125	
Proportion of participants with oxygen desaturation, n (%)			
Day 3	1/139 (0.7)	2/125 (1.6)	0.605
Day 7	2/138 (1.4)	2/124 (1.6)	1.000
Day 14	5/135 (3.7)	1/124 (0.8)	0.216
Change of WHO progression score from baseline, n (%)			

Day 3	0 (-3, 0)	0 (-3, 0)	0.956
Day 7	0 (-4, 0)	0 (-3, 0)	0.946
Day 14	1 (-5, 1)	1 (-2, 1)	0.160
Absence of symptom, n (%)			
Day 3	29/139 (20.9)	21/125 (16.8)	0.434
Day 7	62/139 (44.6)	61/125 (48.8)	0.538
Day 14	102/139 (73.4)	105/125 (84.0)	0.051
Hospitalization due to clinical progression within 14 days, n (%)	4/139 (2.9)	2/125 (1.6)	0.687
Ct ≥20 (n=179)	n=90	n=89	
Proportion of participants with oxygen desaturation, n (%)			
Day 3	1/90 (1.1)	1/89 (1.1)	1.000
Day 7	0/89 (0.0)	2/87 (2.3)	0.243
Day 14	1/89 (1.1)	3/88 (3.4)	0.368
Change of WHO progression score from baseline, n (%)			
Day 3	0 (-3, 0)	0 (-5, 0)	0.327
Day 7	0 (-2, 0)	0 (-5, 0)	0.109
Day 14	1 (-1, 1)	1 (-5, 1)	0.562
Absence of symptom, n (%)			
Day 3	27/90 (30.0)	23/89 (25.8)	0.618
Day 7	56/90 (62.2)	54/89 (60.7)	0.879
Day 14	72/90 (80.0)	71/89 (79.8)	0.559
Hospitalization due to clinical progression within 14 days, n (%)	0/90 (0.0)	2/89 (2.2)	0.246

With or without Favipiravir (FVP)			
With FVP (n=432)	n=223	n=209	
Proportion of participants with oxygen desaturation, n (%)			
Day 3	2/223 (0.9)	3/209 (1.4)	0.676
Day 7	2/221 (0.9)	3/206 (1.5)	0.676
Day 14	6/218 (2.8)	4/207 (1.9)	0.752
Change of WHO progression score from baseline, n (%)			
Day 3	0 (-3, 0)	0 (-5, 0)	0.287
Day 7	0 (-4, 0)	0 (-5, 0)	0.143
Day 14	0 (-2, 1)	1 (-5, 1)	0.288
Absense of symptom, n (%)			
Day 3	52/223 (23.3)	43/209 (20.6)	0.561
Day 7	114/223 (51.1)	114/209 (54.5)	0.500
Day 14	168/223 (75.3)	172/209 (82.3)	0.079
Hospitalization due to clinical progression within 14 days, n (%)	4/223 (1.8)	4/209 (1.9)	1.000
Without FVP (n=11)	n=6	n=5	
Proportion of participants with oxygen desaturation, n (%)			
Day 3	0/6 (0.0)	0/5 (0.0)	
Day 7	0/6 (0.0)	1/5 (20.0)	0.455
Day 14	0/6 (0.0)	0/5 (0.0)	
Change of WHO progression score from baseline			
Day 3	-0.5 (-2, 0)	0 (-1, 0)	0.329
Day 7	-0.5 (-2, 0)	0 (-1, 0)	0.329

Day 14	1 (0, 1)	0 (-2, 0)	0.052
Absence of symptom, n (%)			
Day 3	4/6 (66.7)	1/5 (20.0)	0.242
Day 7	4/6 (66.7)	1/5 (20.0)	0.242
Day 14	6/6 (100.0)	4/5 (80.0)	0.455
Hospitalization due to clinical progression within 14 days (%)	0/6 (0.0)	0/5 (0.0)	
COVID-19 vaccination status			
No vaccination (n=141)	n=81	n=60	
Change of WHO progression score from baseline, n (%)			
Day 3	7 (8.6)	11 (18.3)	0.125
Day 7	7 (8.6)	12 (20.0)	0.079
Day 14	2 (2.5)	5 (8.3)	0.136
Absence of symptom, n (%)			
Day 3	16 (19.8)	10 (16.7)	0.668
Day 7	38 (46.9)	27 (45.0)	0.865
Day 14	63 (77.8)	49 (81.7)	0.675
Incomplete course of vaccine (n=207)	n=99	n=108	
Change of WHO progression score from baseline, n (%)			
Day 3	17/99 (17.2)	19/108 (17.6)	1.000
Day 7	17/99 (17.2)	22/108 (20.4)	0.597
Day 14	4/99 (4.0)	1/108 (0.9)	0.195
Absence of symptom, n (%)			
Day 3	23/99 (23.2)	21/108 (19.4)	0.610
Day 7	49/99 (49.5)	61/108 (56.5)	0.332

Day 14	74/99 (74.7)	87/108 (80.6)	0.403
Complete course of vaccine (n=95)	n=49	n=46	
Change of WHO progression score from baseline, n (%)			
Day 3	9/49 (18.4)	7/46 (15.2)	0.787
Day 7	9/49 (18.4)	6/46 (13.0)	0.578
Day 14	2/49 (4.1)	2/46 (4.3)	1.000
Absence of symptom, n (%)			
Day 3	17/49 (34.7)	13/46 (28.3)	0.517
Day 7	31/49 (63.3)	27/46 (58.7)	0.679
Day 14	37/49 (75.5)	40/46 (87.0)	0.195

Table S4: Factors associated with favorable outcome at Day 7 in treatment study

Variables	Presence of symptom (n=210)	Absence of symptom (n=233)	*P* value
Age (years), Mean (SD)	38.3 (40.4)	40.4 (12.1)	0.068
Gender, n (%)			0.056
Male	81 (38.6)	111 (47.6)	
Female	129 (61.4)	122 (52.4)	
Weight (kg), Mean (SD)	66.9 (15.4)	70.0 (16.7)	0.044
≤90 kg, n (%)	192 (91.4)	210 (90.1)	0.743
>90 kg, n (%)	18 (8.6)	23 (9.9)	
Presence of underlying diseases, n (%)			
Diabetes mellitus	15 (7.1)	16 (6.9)	1.000
Hypertension	22 (10.5)	26 (11.2)	0.879
Dyslipidemia	14 (6.7)	11 (4.7)	0.414
CAD	3 (1.4)	5 (2.1)	0.727
CKD	1 (0.5)	1 (0.4)	1.000
Cirrhosis	1 (0.5)	0 (0.0)	0.474
Chronic lung diseases	1 (0.5)	0 (0.0)	0.474
CVA	1 (0.5)	0 (0.0)	0.474
Cancer	0 (0.0)	1 (0.4)	1.000
Autoimmune disease	1 (0.5)	1 (0.4)	1.000
Others	33 (15.7)	29 (12.4)	0.340
Risk of exposure: Family contact (Know), n (%)	148 (70.5)	162 (69.5)	0.836

Duration of last contact until swab (n=313), n (%)			0.498
≤ 7 days	139 (94.6)	149 (92.0)	
>7 days	8 (5.4)	13 (8.0)	
Duration of illness prior to enrollment (n=394), n (%)			
≤ 3 days	154 (80.2)	146 (73.4)	0.120
>3 days	38 (19.8)	53 (26.6)	
WHO clinical score at baseline, n (%)			
Score 1	17 (8.1)	34 (14.6)	0.037
Score 2	193 (91.9)	199 (85.4)	
Previous vaccination, n (%)			0.079
No	76 (36.2)	65 (27.9)	
Incomplete course of vaccine	97 (46.2)	110 (47.2)	
Complete course of vaccine	37 (17.6)	58 (24.9)	
Medication, n (%)			
Favipiravir	204 (97.1)	228 (97.9)	0.763
Others (Steroid, *Andrographis paniculata*)	0 (0.0)	3 (1.3)	0.250
Receiving study medications, n (%)			0.703
Ivermectin	111 (52.9)	118 (50.6)	
Placebo	99 (47.1)	115 (49.4)	

Supplementary Table S5: Adverse events of study medication separated by prevention or treatment study

Symptoms (Modified-ITT)	Treatment study					Prevention study				
	Ivermectin (n=253)		Placebo (n=272)		p-value	Ivermectin (n=229)		Placebo (n=214)		p-value
	no. events	no. case n (%)	no. events	no. case n (%)		no. events	no. case n (%)	no. events	no. case n (%)	
Total	72	53 (20.9)	67	49 (18.0)	0.440	69	51 (22.3)	77	43 (20.1)	0.642
Ocular problems	23	22 (8.7)	0	0 (0.0)	<0.001	5	5 (2.2)	4	3 (1.4)	0.725
Diarrhea	9	9 (3.6)	11	10 (3.7)	1.000	14	14 (6.1)	10	9 (4.2)	0.399
Myalgia	7	6 (2.4)	7	7 (2.6)	1.000	8	7 (3.1)	12	10 (4.7)	0.461
Headache	1	1 (0.4)	8	8 (2.9)	0.039	9	8 (3.5)	17	14 (6.5)	0.189
Neurologic ADR	2	2 (0.8)	2	2 (0.7)	1.000	6	6 (2.6)	9	8 (3.7)	0.592
Rash	3	3 (1.2)	3	3 (1.1)	1.000	4	4 (1.7)	1	1 (0.5)	0.374
Nausea/ vomiting	1	1 (0.4)	5	5 (1.8)	0.218	5	5 (2.2)	7	6 (2.8)	0.765
Pruritus	1	1 (0.4)	2	2 (0.7)	1.000	0	0 (0.0)	1	1 (0.5)	0.483
Others	25	23 (9.1)	29	29 (10.7)	0.562	18	17 (7.4)	16	15 (7.0)	1.000
