# Supplementary figures and images for: Safety and Efficacy of Ivermectin for the Prevention and Treatment of COVID-19: A Double-Blinded Randomized Placebo-Controlled Study
Source: Antibiotics (Basel). 2022 Jun 12;11(6):796. doi: 10.3390/antibiotics11060796 (PMC9219629; doi:10.3390/antibiotics11060796)

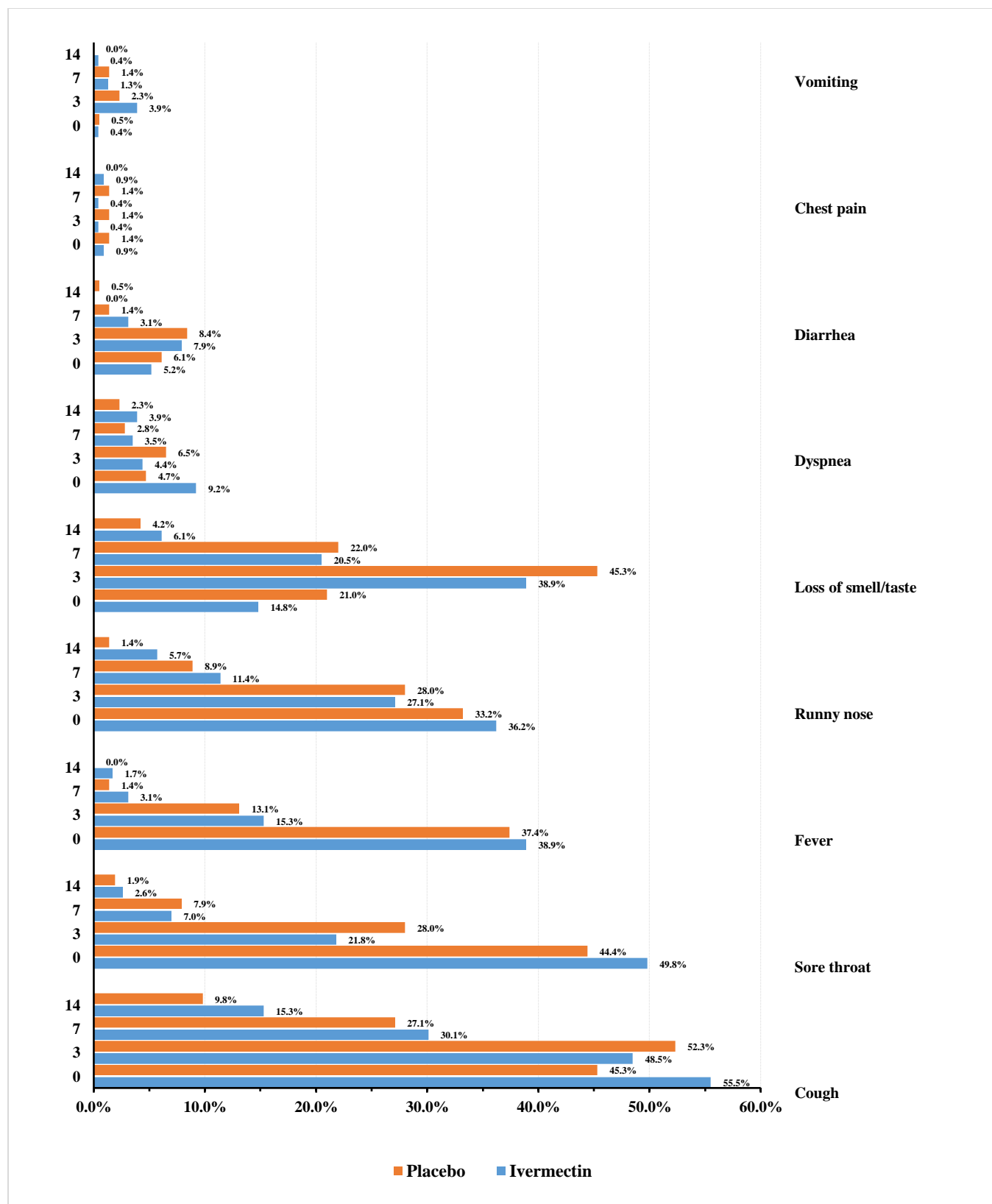

Supplement: Supplementary file 1 [file antibiotics-11-00796-s001.zip › Supplementary Figure S1_Ivermectin.pdf]
